# Network Analysis for Better Understanding the Complex Psycho-Biological Mechanisms behind Fibromyalgia Syndrome

**DOI:** 10.3390/diagnostics12081845

**Published:** 2022-07-30

**Authors:** Juan Antonio Valera-Calero, Lars Arendt-Nielsen, Margarita Cigarán-Méndez, César Fernández-de-las-Peñas, Umut Varol

**Affiliations:** 1 VALTRADOFI Research Group, Department of Physiotherapy, Faculty of Health, Universidad Camilo José Cela, 28692 Villanueva de la Cañada, Spain; javalera@ucjc.edu (J.A.V.-C.); 1umutvarol7@gmail.com (U.V.); 2 Department of Physiotherapy, Faculty of Health, Universidad Camilo José Cela, 28692 Villanueva de la Cañada, Spain; 3 Center for Neuroplasticity and Pain (CNAP), Sanse-Motorisk Interaktion (SMI), Department of Health Science and Technology, Faculty of Medicine, Aalborg University, 9220 Aalborg, Denmark; lan@hst.aau.dk; 4 Department of Medical Gastroenterology, Mech-Sense, Aalborg University Hospital, 9000 Aalborg, Denmark; 5 Department of Psychology, Universidad Rey Juan Carlos, 28922 Alcorcón, Spain; margarita.cigaran@urjc.es; 6 Department of Physical Therapy, Occupational Therapy, Rehabilitation and Physical Medicine, Universidad Rey Juan Carlos, 28922 Alcorcon, Spain

**Keywords:** network analysis, fibromyalgia, pain, function, clinical decision rules

## Abstract

The aim of this study was to assess potential associations between sensory, cognitive, health-related, and physical variables in women with fibromyalgia syndrome (FMS) using a network analysis for better understanding the complexity of psycho-biological mechanisms. Demographic, clinical, pressure pain threshold (PPT), health-related, physical, and psychological/cognitive variables were collected in 126 women with FMS. A network analysis was conducted to quantify the adjusted correlations between the modeled variables and to assess the centrality indices (i.e., the degree of connection with other symptoms in the network and the importance in the system modeled as a network. This model showed several local associations between the variables. Multiple positive correlations between PPTs were observed, being the strongest weight between PPTs over the knee and tibialis anterior (ρ: 0.28). Catastrophism was associated with higher hypervigilance (ρ: 0.23) and lower health-related EuroQol-5D (ρ: −0.24). The most central variables were PPT over the tibialis anterior (the highest strength centrality), hand grip (the highest harmonic centrality) and Time Up and Go (the highest betweenness centrality). This study, applying network analysis to understand the complex mechanisms of women with FMS, supports a model where sensory-related, psychological/cognitive, health-related, and physical variables are connected. Implications of the current findings, e.g., developing treatments targeting these mechanisms, are discussed.

## 1. Introduction

Fibromyalgia syndrome (FMS) is a chronic pain condition that affects 6.6% of the general population [1]. In Spain, where the prevalence is considerably lower (2.4%), FMS involves an economic burden of EUR 12.993 billion annually [1]. FMS is associated with a plethora of sensory and motor symptoms ranging from widespread pain, sleep disturbances, cognitive dysfunction, anxiety, and depression to fatigue and stiffness [2]. The complexity of its underlying mechanisms has led to FMS being considered a central sensitivity syndrome [3], and, more recently, a nociplastic condition [4].

There is evidence supporting the presence of sensitization mechanisms in FMS [5,6]. In addition, cognitive and psychological factors such as hypervigilance, catastrophism, and kinesiophobia have also been found to be involved in severity [7] and physical function [8] in women with FMS. Similarly, women with FMS also exhibit generalized muscle weakness and decreased physical capacity [9]. In fact, it has recently been observed that functional capacity, muscular strength, pain threshold, and anxiety are factors predicting health-related quality of life in women with FMS [10]. It seems that clinical, cognitive, sensory, and physical factors are interconnected in complex mechanisms explaining the symptomatology experienced by these patients [11,12].

Previous studies have investigated the association between pain, related disability, physical, and psychological variables in women with FMS used Pearson Product–Moment Correlations and linear regression analyses [7,8,9,10,11,12]. Pearson Product–Moment Correlation ignores the potential for pairwise associations arising from interaction with another variable (e.g., a common cause), whereas linear regression ignores the possibility of bidirectional relationships since the researcher is constrained to modeling the unidirectional relationship of the independent variables on the dependent variable [13]. Network analysis provides a novel methodology to understand complex relationships addressing the aforementioned limitations [14] and provides a method to identify the most important variables within the identified network [15]. From a network perspective, FMS emerges and is sustained by a collection of reciprocal interactions between clinical, psychological, cognitive, and physical systems. Some recent studies have used network analysis to better understand the complexity of chronic pain [16,17]; however, no previous study has used network analysis in individuals with FMS. Since an ideal theoretical framework of FMS integrates reciprocal interactions between biology (sensory and physical aspects) and behaviors (psychological and cognitive aspects) [18], we applied network analysis to better understand the interactions between sensory, cognitive, health-related, and physical variables in women with FMS. Accordingly, the main aims of this study were to (1) describe a network including demographic, clinical, psycho-physical, psychological, health-related, and physical variables in women with FMS, and (2) illustrate the potential of a network analysis perspective for understanding complex mechanisms of FMS, generating research questions, and improving potential treatment strategies.

## 2. Materials and Methods

### 2.1. Participants

Women with a medical diagnosis of FMS [19,20] were recruited from those responding to local announcements, at AFINSYFACRO and FIBROPARLA Fibromyalgia Association, Madrid, Spain. They were excluded if presenting with previous whiplash injury, previous surgery, neuropathic conditions (e.g., radiculopathy or myelopathy), other underlying medical conditions (e.g., tumor), or regular medication use affecting muscle tone or pain perception, except use of non-steroidal anti-inflammatory drugs (NSAIDs) sporadically (maximum twice a week). The Local Ethics Committee of Universidad Rey Juan Carlos approved the study (URJC 08-30-2020). All participants signed written informed consent prior to their inclusion in the study.

### 2.2. Clinical Variables

Participants rated their mean pain intensity at rest, the worst pain intensity at rest, and their pain intensity experienced during daily living activities on three different 11-point numerical point-rating scales (NPRS; 0: no pain; 10: maximum pain) [21]. Participants completed a pain drawing by shading, with a red pencil, the location of their symptoms on a ventral and a dorsal paper body chart. Pain extent, reported as the total number of pixels in the digital pain drawings, was computed [22]. Pain extent was expressed as a percentage of the body chart area (ventral: 476,650 pixels, dorsal: 489,592 pixels).

### 2.3. Psycho-Physical Variables

**Figure 1:**
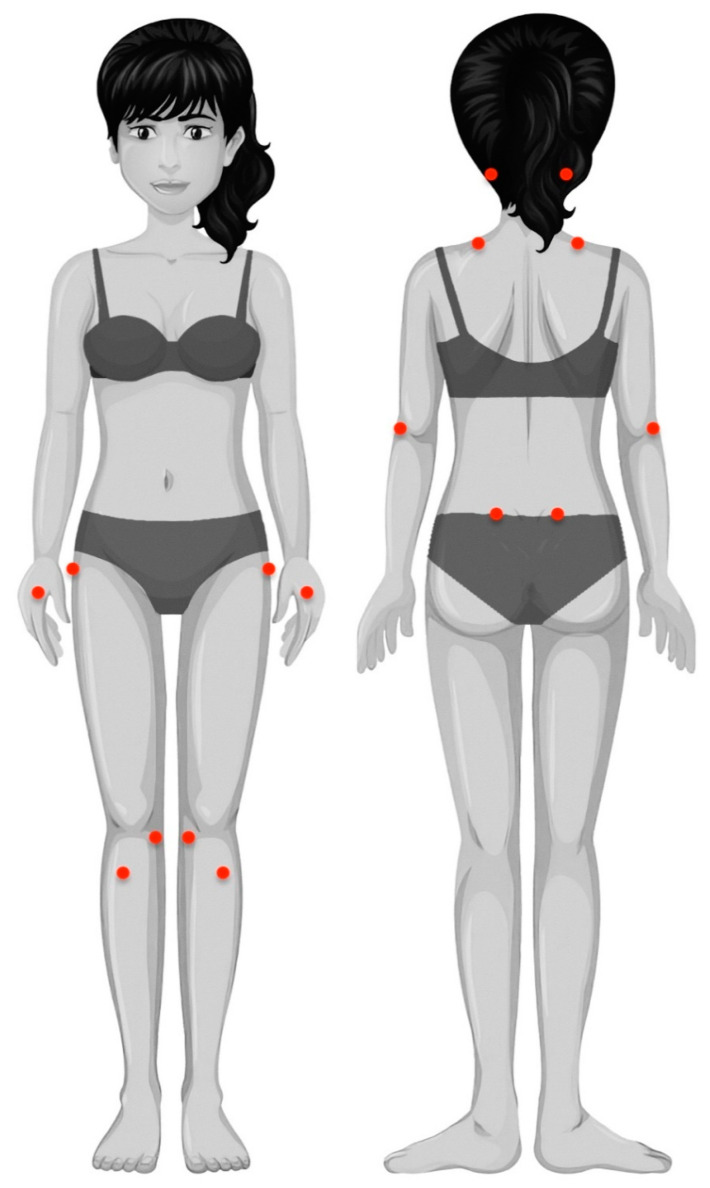
Location of pressure pain threshold measurements: mastoid process, upper trapezius muscle, lateral epicondyle, second metacarpal, posterosuperior iliac spine, greater trochanter, pes anserine, and tibialis anterior muscle.

Widespread pressure pain thresholds (PPTs) were assessed with an electronic algometer (Somedic^®^, Sollentuna, Sweden) in the following areas: mastoid process, upper trapezius muscle, elbow, hand, posterosuperior iliac spine, greater trochanter, knee, and tibialis anterior, as illustrated in Figure 1 [23]. Pressure was applied at a rate of approx. 30 kPa/s on each point. The mean of 3 trials on each point, with a resting period of 30 s between each, was calculated and used in the analysis. This testing procedure showed good reliability (ICC ≥ 0.88) in patients with FMS [24]. Since no side-to-side differences were found at any assessed point (independent student *t*-tests), the mean of both sides was used in the analysis.

### 2.4. Psychological/Cognitive Variables

Pain hypervigilance was assessed with the short-form 9-item Spanish Pain Vigilance and Awareness Questionnaire (PVAQ), since this questionnaire is a valid and reliable tool to identify ideas of observing, monitoring, and focusing on pain in FMS [25].

The Spanish version of the Pain Catastrophizing Scale (PCS) is a 13-item self-reported questionnaire commonly used to assess pain catastrophizing responses in individuals with pain [26]. Items are answered in a 5-point Likert scale where 0 means “never” and 4 means “always” (total score 0.52). This self-reported questionnaire evaluates rumination (constant worry and inability to inhibit thoughts related to pain), magnification (exaggeration of unpleasantness of painful situations and expectations of negative consequences), and despair (inability to face pain) aspects [26].

### 2.5. Health-Related Variables

The Spanish version of the fibromyalgia impact questionnaire (FIQ) was used to assess related disability [27]. It includes 10 subscales assessing the daily tasks function, number of days feeling good during the last 7 days, the interference of FMS with their work, pain intensity, fatigue, night resting, stiffness, anxiety, and depression. The final score ranges from 0 to 100, where higher scores involve greater disability and severity [27].

The Fibromyalgia Health Assessment Questionnaire (FHAQ) is a disease-specific tool used for assessing functional ability in FMS in a single questionnaire with 8 items with scores ranging from 0 to 3 [28]. The FHAQ final score is calculated as the mean of the 8 items, where lower scores (0) mean less difficulty during daily functional activities.

Health-related quality of life was assessed with the paper-based five-level version of the EuroQol-5D questionnaire [29]. The EuroQol-5D includes five descriptive health dimensions (mobility, self-care, daily activities, pain, and depression/anxiety) ranging from 1 (no problems) to 3 (severe problems). Responses were converted into a single index number between 0 and 1, where 0 corresponds to a health state judged to be equivalent to death and 1 corresponds to optimal health, by applying crosswalk index values for life in Spain [30].

### 2.6. Physical Variables

Hand-grip maximum force was bilaterally assessed with a Jamar hand dynamometer (JLW Instruments, Chicago, IL, USA). The examiner explained and demonstrated the testing procedure before data collection. Each subject placed the Jamar in their hand, with the arm beside the trunk, the shoulder in a neutral position, and the elbow flexed at 90°, and pulled the metal bar with their fingers [31]. The mean of three trials for each hand (with 3 min resting periods between repetitions) was calculated. The reliability of hand-grip force in individuals with FMS has been shown to be excellent [31].

The Timed Up and Go (TUG) test is an easy, cost-effective, rapid, and valid tool providing valuable predictive information to identify patients with a high risk of falls [32]. The patient is placed in sitting position in an armchair and is asked to stand up without the use of the arms, to walk at a comfortable and safe speed up to a line placed 3 m from the chair, to turn back to the chair, and to sit down again. The TUG has shown to be a reliable physical fitness test for assessing agility/dynamic balance in women with FMS [33].

### 2.7. Approach to Network Analysis

#### 2.7.1. Software and Packages

The R software v.4.1.1 for Windows 10 was used for the data analysis [34]. Furthermore, qgraph (v.1.6.9) [35], glasso (v.1.11) [36], CINNA (v.1.1.54) [37], igraph (v.1.2.6) for [38], huge (v.1.3.5) [39], missForest (v.1.4) [40], and bootnet (v.1.4.3) [41] packages were used.

#### 2.7.2. Missing Value Imputation

Exploratory data analysis conducted on the dataset revealed missing values in 26 parameters. Removal of the missing values would result in a loss of 15.1% of the data (19 records), which would reduce sample size and may introduce bias [42]. As missing data not collected did not depend on any other variable according to the study design, i.e., missing completely at random (MCAR), data imputation was performed using missForest [43,44,45].

#### 2.7.3. Network Estimation

Networks are used to represent and explore complex systems by using nodes (vertices) and edges. The nodes were made up of the 26 variables collected and included as continuous. Edges constitute the link connecting the nodes and are interpreted as “the remaining association between two nodes after controlling for all other information possible” [13]. Therefore, edges represent the regularized partial correlations in terms of magnitudes and direction with weights and colors, respectively. The width (thickness) and the color saturation of the edge indicates the magnitude of the association between two nodes; and the color of the edge indicates the direction of the partial correlation, where red lines refer to negative and green lines refer to positive partial correlations.

A nonparanormal transformation was applied over the complete dataset after the missing data imputation to ensure 26 variables (y) were multivariate normally distributed [39], which is a requirement for Gaussian Graphical Model (GGM) estimation [41]. For the network estimation, the graphical least absolute shrinkage and selection operator (LASSO) regularization LASSO was used to draw out a sparse model.

Since LASSO seeks to maximize specificity (aiming to include as few false positives as possible), the estimated network ends up being sparse, i.e., including fewer edges compared to a saturated model [36], which makes the model easier to interpret [13]. Selection of the LASSO tuning parameter was performed by minimization of the Extended Bayesian Information Criterion (EBIC) since it has been shown to perform well in retrieving the true network structure, featuring high specificity (i.e., not including edges that are not in the true network) but varying sensitivity (i.e., estimating edges that are existent in the true network) based on the true network structure and sample size [13].

#### 2.7.4. Node Centrality

Node centrality is used predict several network processes, including the amount of flow traversing a node or tolerance of the network to the removal of selected nodes, and constitutes a guide for network interventions [46]. Node centrality was estimated based on the strength, harmonic, and betweenness centrality [13,47].

Strength centrality is a blunt measure that takes a node’s total level of involvement in the network and not the number of connections with other nodes. Therefore, using other centrality indicators is important to derive accurate conclusions [48,49,50].

Since closeness centrality cannot be calculated for the unconnected nodes found in this study [46], harmonic centrality was assessed. This estimate provides information about whether a node influence can reach other nodes more quickly than other peripheral ones due to the shortest paths connecting itself and other nodes [34,51,52].

Finally, betweenness centrality can be interpreted as the percentage of shortest paths that must go through the target node. Therefore, a node with a high betweenness centrality would act as an intermediary in the transmission of information or resources between other nodes or even clusters of nodes in the network [52].

#### 2.7.5. Network Edge and Node Centrality Variability

Edge weight and centrality index variability were assessed by using 2000 iterations to bootstrap 95% confidence intervals (CIs) of edge weights [53]. Wide confidence intervals would entangle the interpretation of the edge strength, yet not the presence since model selection is already performed by LASSO. In addition, it should be noted that the sign of the edge can be interpreted independently of the CI width, as LASSO rarely retains an edge that can be positive or negative in the model.

For assessing the variability of the centrality indices (CS-coefficient), a participant-dropping subset bootstrap was utilized [53]. This approach drops a percentage of participants and re-estimates the network and the three related centrality indices. The CS coefficient (correlation stability) reflects the maximum proportion of data that can be dropped (ideally >0.25 [13,52]) to retain a correlation >0.7 with the original centrality indices (95% certainty) [53].

#### 2.7.6. Community Detection

Some nodes (variables) often form distinct groups where there are many relations in between compared to the others in the system [32]. In network analysis, community detection is the process of identifying these relatively dense clusters of nodes [54], which constitutes a data-clustering problem. In this study, the Louvain community detection algorithm was utilized for identifying non-overlapping communities since it iteratively uses modularity to optimize its partitions [54,55].

## 3. Results

**Figure 2:**
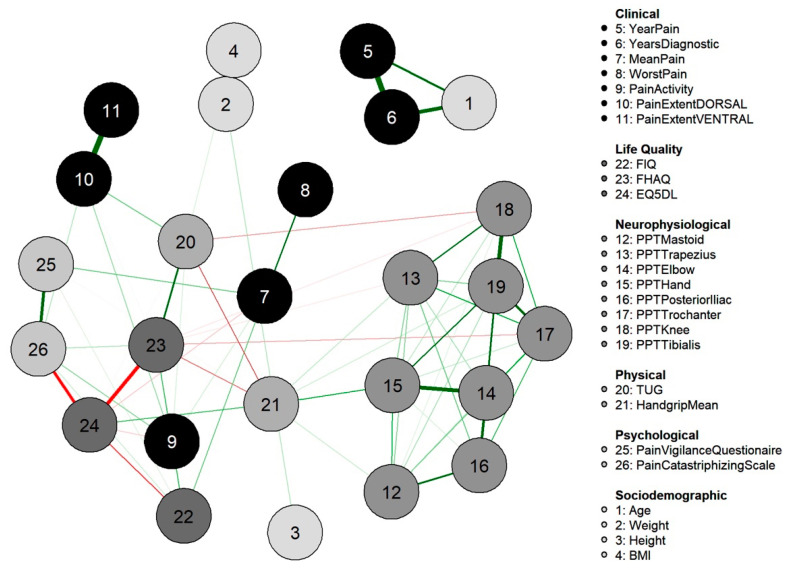
Network analysis of the association between demographic, clinical, cognitive, psycho-physical, health-related, and physical measures. Edges represent connections between two nodes and are interpreted as the existence of an association between two nodes, adjusted for all other nodes. Each edge in the network represents either positive regularized adjusted associations (green edges) or negative regularized adjusted associations (red edges). The thickness and color saturation of an edge denotes its weight (the strength of the association between two nodes).

Table 1 summarizes descriptive statistics of the variables before and after missing value imputation, included in the network analysis. Although there were no racial/ethnic restrictions, all participants (*n* = 126) were white women. The network obtained is displayed in Figure 2 and shows partial correlations between sociodemographic, clinical, psychophysical, cognitive/psychological, health-related, and physical variables. Several correlations between PPTs were observed among the assessed points (nodes 12 to 19), with a correlation (ρ) between PPTs over the knee and tibialis anterior (nodes 18 and 19) of 0.28, and between the elbow and hand (nodes 14 and 15) of 0.27 (Figure 2). In addition, PPTs were also associated with the physical variable of hand grip (node 21), with the highest correlation (ρ: 0.15) with PPT at the hand (node 15). Regarding psychological/cognitive variables, catastrophism was associated with higher hypervigilance (ρ: 0.23) and lower health-related EuroQol-5D (ρ: −0.24). The impact on health-related quality had multiple correlations with the physical and psychological/cognitive variables, but not clinical, sociodemographic, or PPT (Figure 2).

**Figure 3:**
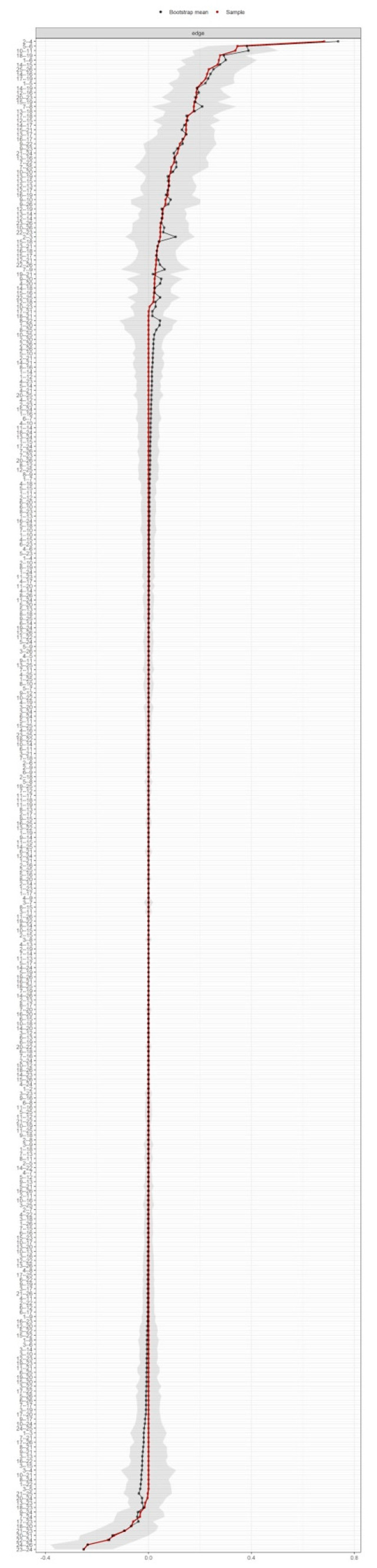
Bootstrapped 95% quantile confidence interval of the estimated edge weights of the network. “Bootstrap mean” reflects the average magnitude of edge weights across the bootstrapped samples. “Sample” reflects the magnitude of edge weights of the original network built on the entire input dataset.

The variability associated with the weight of each edge is depicted graphically in Figure 3. The non-overlap of the 95% CI of the edge between PPTs at the lateral epicondyle and posterior iliac crest locations (nodes 14 and 16) with the 95% CI of the edge between pain during daily living activities and FHAQ (nodes 9 and 23) indicates that the strength of former is significantly greater than the latter.

**Figure 4:**
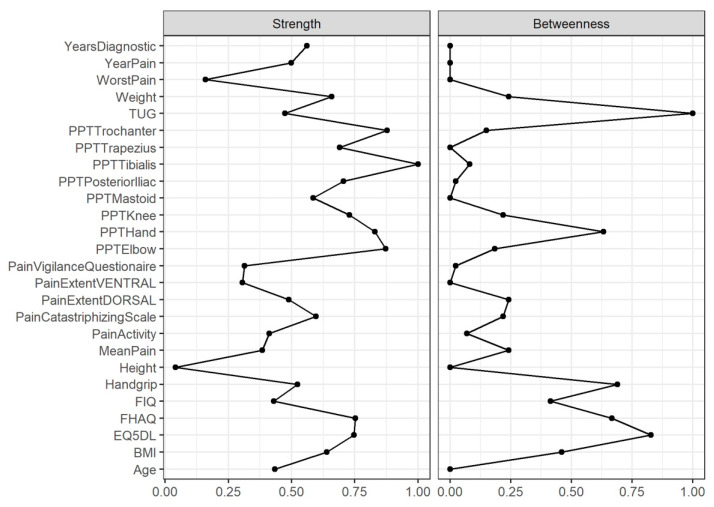
Centrality measures of strength and betweenness of each node in the network. A centrality value of 1 indicates maximal importance, and 0 indicates no importance.

**Figure 5:**
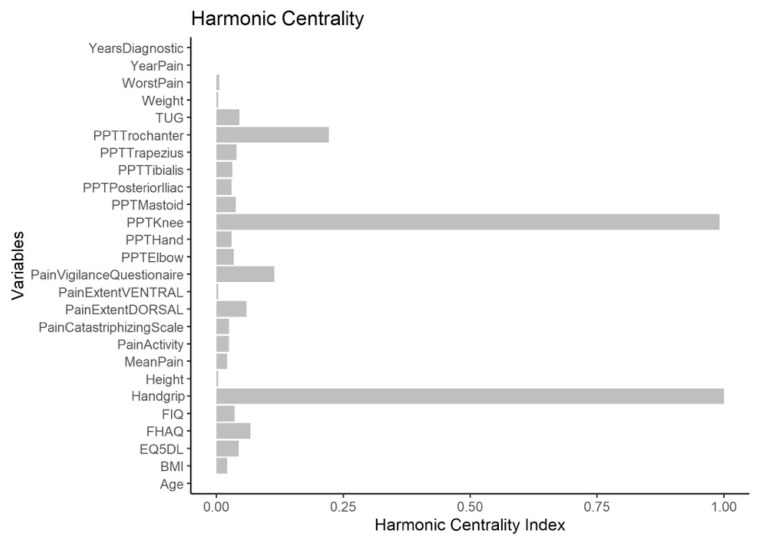
Harmonic centrality measure of each node in the network. A centrality value of 1 indicates maximal importance, and 0 indicates no importance.

**Figure 6:**
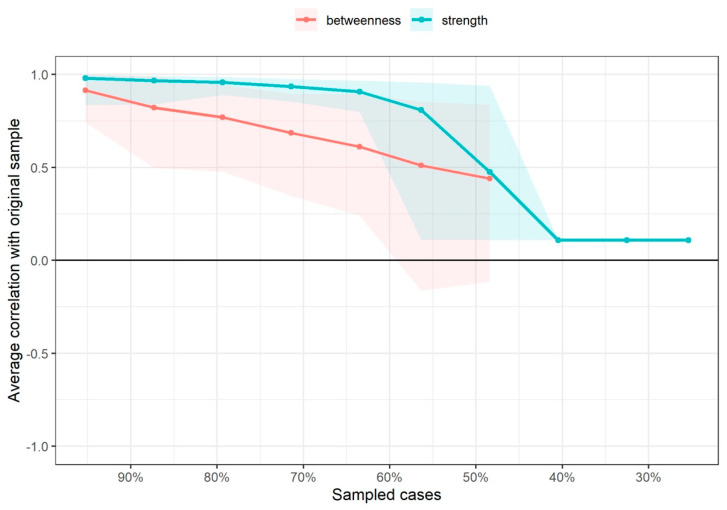
Average correlations between centrality indices of networks sampled with persons dropped and networks built on the entire input dataset at all follow-up time points. Lines indicate the means and areas indicate the range from the 2.5th quantile to the 97.5th quantile.

The node with the highest strength centrality was PPT at the tibialis anterior muscle, followed by PPTs at the lateral epicondyle and greater trochanter locations (Figure 4). The node with the highest harmonic centrality was hand-grip force, followed by the PPT at the knee (Figure 5). Finally, the node with the highest betweenness centrality was TUG, followed by the EQ5DL (Figure 4). The betweenness and strength measures of the network were extremely unstable at CS_cor=0.7_ = 0.048 and CS_cor=0.7_ = 0.365, respectively. The closeness centrality measure could not be assessed with bootstrapping since the resulting networks were unconnected (Figure 6).

**Figure 7:**
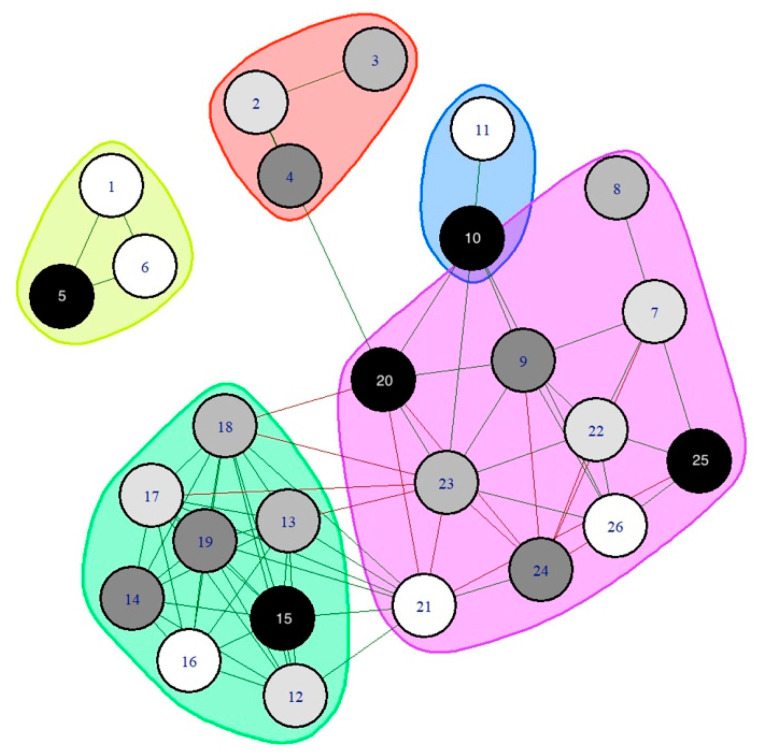
Clusters identified by the Louvain community detection algorithm. Green cluster: psycho-physical (PPTs) variables; purple cluster: pain-related, cognitive, health-related, and physical variables; blue cluster: pain extent variables; red cluster: sociodemographic variables; yellow cluster: diagnostic variables. Numbers represent the same nodes reported in Figure 2.

Parallel to the visualization of the network, five clusters were identified by the Louvain community detection algorithm (Figure 7). Most of the nodes with the same variable classification ended up in the same cluster, e.g., all PPTs grouped in the green cluster, and cognitive, health-related, and physical variables in the purple cluster (Figure 7).

## 4. Discussion

This is the first study applying network analysis to better understand the associations behind sensory and motor symptoms associated with FMS. Consistent with current theories, the network supports a model where pain-related, psycho-physical, cognitive, health-related, and physical variables are interconnected in women with FMS from a clinical point of view. In fact, the modeled associations revealed one cluster grouping psycho-physical (neurophysiological) variables and a second one grouping pain-related, cognitive, health-related, and physical variables.

The psycho-physical cluster grouped all widespread PPTs, whereas the second one grouped pain-related, cognitive, health-related, and physical variables. Interestingly, the associations between clusters were small in the network supporting psychophysical variables, such as widespread pressure sensitivity, representing a mechanism process not directly associated with clinical and functional repercussions. In line with these results, it has been previously reported that PPTs are not linearly associated with clinical outcomes [56]. This lack of association could be related to the fact that PPTs are used for assessing pressure pain sensitivity as a manifestation of hyperalgesia and excitability of the central nervous system [5,6], whereas pain and related functions are clinical variables explained by complex biopsychosocial interactions. Given the small associations between both clusters, the current findings support the need to collect subjective and objective outcomes in women with FMS to obtain a holistic understanding of the disease. In fact, the relevance of assessing objective and subjective variables in women with FMS has been previously recommended [8]. The inclusion of psychophysical and patient-reported outcome measures (PROMs) could provide a more holistic view of the condition.

The network also revealed that those edges with the strongest weights were PPTs at the knee and tibialis anterior muscle and PPTs at the elbow and hand supporting the presence of widespread pressure pain sensitivity as a clinical manifestation of central sensitization, as commonly reported in the literature [5,6]. Furthermore, PPTs at the tibialis anterior muscle, lateral epicondyle, and greater trochanter showed the highest strength centrality measures, meaning that these nodes influence other nodes (or are influenced by them) directly. In addition, physical variables such as TUG and EQ5DL also showed the highest betweenness centrality. These results suggest that if clinicians want to influence other variables, e.g., pain-related, cognitive, or physical variables, the best variable to focus treatment on would be to influence widespread pressure pain sensitivity, that is, central sensitization, and physical capacity, as reflected by the TUG. Interestingly, PPTs at the hand were associated with hand-grip force, reflecting that pressure sensitivity is able to influence physical function. These hypotheses support why exercise, a therapeutic strategy able to reduce pain sensitivity throughout adaptations in the central nervous system [57], has the highest level of evidence for the management of FMS patients [58]. The application of progressive exercise programs could lead to a desensitization of the nervous system, which would lead to a decrease in generalized pressure pain hyperalgesia, i.e., exercise-induced analgesia [57]. Additionally, since aerobic and strengthening exercise programs are those with the highest level of evidence in FMS [58], their application would also improve related functions. Nevertheless, it is important to consider that specific pain mechanisms underpinning each pain condition must be considered to optimize exercise program prescription in people with chronic conditions of nociplastic predominance, e.g., FMS [59]. For instance, the application of an inappropriate exercise with high load could worsen symptomatology in women with FMS with highly nociplastic predominance.

The network also revealed that cognitive variables such as catastrophism were associated with health-related variables (EuroQol-5D). Our results agree with those previously reported by Estévez-López et al. [8], who found that high catastrophizing promotes a feeling of reduced ability to do daily living activities, affecting health-related quality of life. In agreement with our results, a recent meta-analysis found that psychological factors and physical activity levels are associated with somatosensory function in individuals with joint pain [60]. In fact, evidence suggests that improving cognitive aspects such as fear avoidance beliefs is crucial for positive effects on physical activity [61]. Accordingly, this network supports the presence of complex interactions between pain-related, cognitive, and physical variables in FMS. These results further reinforce theories suggesting that management of individuals with FMS should include multimodal therapeutic approaches targeting pain mechanisms (e.g., pain education or physical therapy), cognition (i.e., copying strategies), and physical capacity (i.e., exercise) [62]. In fact, ignoring the management of maladaptive behaviors could decrease the effectiveness of other therapeutic strategies such as exercise.

Although this is the first study using a network analysis in FMS, some limitations should be recognized. Since only women were included, the current findings should not be extrapolated to men with FMS. Conditional independence relationships, as encoded by the edge weights in the networks, cannot be a source of confirmatory causal inference, but may provide indicative potential causal pathways [13]. For example, if all relevant variables are modeled in a network, an observed adjusted association between two variables would only be possible if one variable causes the other, both variables exhibit a bidirectional relationship, or both have a common effect [13]. In other words, biological plausibility between the identified variables is needed. All the modeled variables identified in the current network fulfill this assumption.

## 5. Conclusions

The application of network analysis in women with FMS revealed the presence of two clusters, one grouping mechanistic aspects (including PPTs) and one grouping clinical aspects (including pain-related, cognitive, health-related, and physical variables). The associations between both clusters were small. The network also showed that widespread PPTs, as a sign of central sensitization, but also physical variables, i.e., TUG, were the nodes with the highest centrality measures. Our findings support a model where neurophysiological variables and clinical, sensory, and functional variables are connected but in separate clusters.

## Figures and Tables

**Table 1:** Values (mean ± standard deviation) of demographic, clinical, sensory-related, psychological, and psychophysical variables of the total sample (*n* = 126).

Variable	Pre-Imputation Statistics	Missing Values (*n*; %)	Post-Imputation Statistics
Age (years)	52.2 ± 10.7	1; 0.79	52.2 ± 10.7
Weight (kg)	71.4 ± 16.6	1; 0.79	71.4 ± 16.5
Height (cm)	1.6 ± 0.1	3; 2.38	1.6 ± 0.1
BMI (kg/cm^2^)	27.5 ± 6.2	3; 2.38	27.5 ± 6.1
Years with pain	20.1 ± 15.3	6; 4.76	20.4 ± 14.6
Years with diagnosis	10.2 ± 8.9	3; 2.38	10.1 ± 8.8
Mean pain (NPRS, 0–10)	6.4 ± 1.7	2; 1.58	6.4 ± 1.6
Worst pain (NPRS, 0–10)	7.3 ± 2.2	2; 1.58	7.3 ± 2.1
Pain with activity (NPRS, 0–10)	8.1 ± 1.9	3; 2.38	8.1 ± 1.8
Pain extent dorsal (%)	31.4 ± 26.3	0; 0	31.4 ± 26.3
Pain extent ventral (%)	26.6 ± 25.0	1; 0.79	26.8 ± 25.1
PPT mastoid (kPa)	151.2 ± 90.8	2; 1.59	150.8 ± 90.1
PPT trapezius (kPa)	125.6 ± 60.4	2; 1.59	126.1 ± 60.1
PPT elbow (kPa)	149.0 ± 87.1	2; 1.59	148.7 ± 86.5
PPT hand (kPa)	120.2 ± 59.1	1; 0.79	120.3 ± 58.9
PPT posterior iliac crest (kPa)	233.9 ± 130.7	1; 0.79	233.5 ± 130.3
PPT greater trochanter (kPa)	257.7 ± 123.9	1; 0.79	257.8 ± 123.4
PPT knee (kPa)	148.1 ± 107.1	2; 1.59	149.1 ± 106.8
PPT tibialis anterior (kPa)	187 ± 108.7	3; 2.38	187.9 ± 107.6
Test Up and Go (TUG, s)	12.4 ± 4.9	0; 0	12.4 ± 4.9
Hand grip (kg)	16.7 ± 6.2	9; 7.14	16.1 ± 5.7
FIQ (0–100)	64.8 ± 12.7	0; 0	64.8 ± 12.7
FHAQ (0–3)	1.3 ± 0.6	0; 0	1.3 ± 0.6
EQ5DL (0–1)	0.4 ± 0.3	0; 0	0.4 ± 0.3
PVAQ (0–45)	27 ± 8.2	0; 0	27 ± 8.2
PCS (0–52)	22.5 ± 12.3	0; 0	22.5 ± 12.3

NPRS: Numerical Pain-Rating Scale; PPT: pressure pain thresholds; FIQ: Fibromyalgia Impact Questionnaire; FHAQ: Fibromyalgia Health Assessment Questionnaire; PCS: Pain-Catastrophizing Scale; PVAQ: Pain Vigilance and Awareness Questionnaire.

## Data Availability

Not applicable.
